# Inclisiran SiRNA therapy for durable LDL-C reduction: a systematic review and meta-analysis highlighting a breakthrough in long-term cardiovascular risk management

**DOI:** 10.1186/s12872-026-05515-3

**Published:** 2026-01-10

**Authors:** Shakta Mani Satyam, Mohamed El-Tanani, Mohamed Anas Patni, Abdul Rehman, Sara Muhammad Irshad, Reem Raheem, Esha Junais, Ashika Anu John, Rena Yusuf Rassal, Rashmi Kumari, Sainath P

**Affiliations:** 1 Department of Pharmacology, Translational Medical Research Centre & Central Animal Research Facility, RAK College of Medical Sciences, RAK Medical and Health Sciences University, Ras Al Khaimah, United Arab Emirates; 2 RAK College of Pharmacy, Ras Al Khaimah Medical and Health Sciences University, Ras Al Khaimah, 11172 United Arab Emirates; 3 Department of Community Medicine, RAK College of Medical Sciences, RAK Medical and Health Sciences University, Ras Al Khaimah, United Arab Emirates; 4 Department of Pathological Sciences, College of Medicine, Ajman University, Ajman, United Arab Emirates; 5 A. J. Institute of Hospital Management, A. J. Hospital & Research Centre, Mangalore, India; 6 Department of Perfusion Technology, Manipal College of Health Professions, Manipal Academy of Higher Education, Udupi, Manipal India

**Keywords:** Inclisiran, LDL cholesterol reduction, PCSK9 inhibition, Lipid-lowering agents, Cardiovascular risk management, SiRNA therapy

## Abstract

**Background:**

Inclisiran is a novel small interfering RNA therapeutic developed to lower lipid levels by targeting hepatic production of proprotein convertase subtilisin/kexin type 9. Unlike monoclonal antibodies, Inclisiran silences messenger RNA expression, resulting in sustained reductions in low-density lipoprotein cholesterol with infrequent dosing. This study aimed to systematically evaluate the efficacy and clinical applicability of Inclisiran across randomized controlled trials.

**Methods:**

A systematic literature search was conducted in PubMed, Scopus, Embase, and Cochrane CENTRAL databases up to December 2024. This meta-analysis updates the global evidence base by incorporating 14 randomized trials (> 13,000 participants) identified through December 2024. Beyond earlier syntheses, it provides pooled analyses of multiple lipid parameters, phenotype-stratified subgroup and sensitivity analyses, and meta-regression for baseline LDL-C and statin use, thereby offering the most comprehensive and clinically differentiated evaluation of inclisiran to date. The primary outcome was the mean change in low-density lipoprotein cholesterol levels in patients treated with Inclisiran compared to placebo. Data was pooled using a random-effects model. Heterogeneity was assessed using the I² statistic, and publication bias was evaluated with funnel plot analysis.

**Results:**

Inclisiran treatment resulted in a significant mean reduction of 44.9% in low-density lipoprotein cholesterol levels compared to placebo (95% confidence interval: 39.54% to 50.25%; *p* < 0.001). While statistical heterogeneity was high (I² = 90.3%), visual assessment of the funnel plot suggested minimal risk of publication bias. Subgroup analyses supported consistent efficacy across patient populations, including those with statin intolerance or poor adherence to conventional therapies.

**Conclusions:**

Inclisiran provides a durable, twice-yearly injectable option for lipid management, offering substantial reductions in low-density lipoprotein cholesterol. Its unique RNA-based mechanism and long-acting profile make it particularly suitable for patients who struggle with adherence or have limited response to traditional lipid-lowering medications. These findings support Inclisiran’s role as a promising addition to current strategies for cardiovascular risk reduction.

**Graphical abstract:**

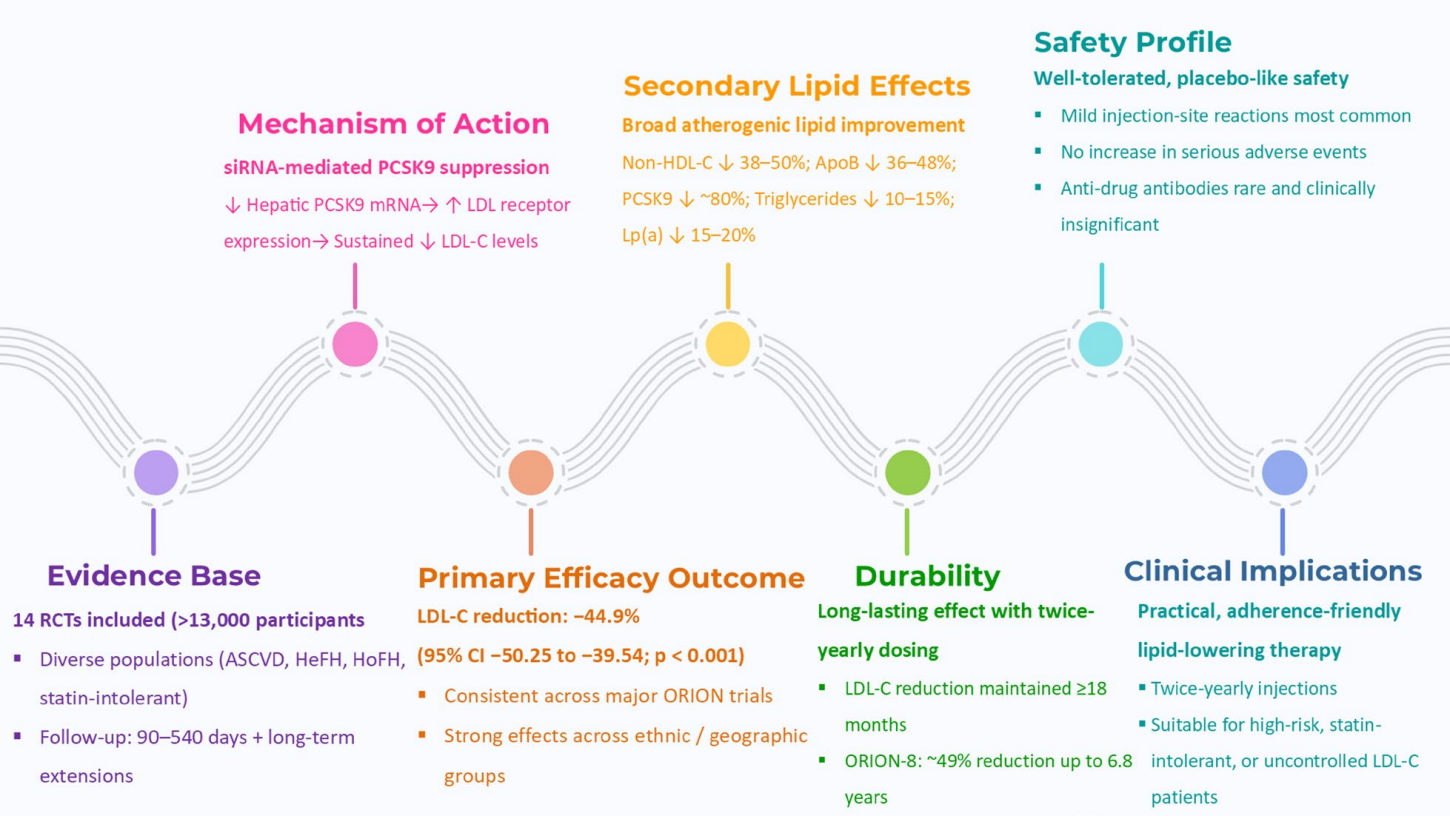

## Introduction

 During the mid-20th century, many high-income countries experienced a sharp rise in cardiovascular disease (CVD) incidence and mortality. However, from the 1960 s onward (late 20th century period), the implementation of effective public health measures, improved diagnostic strategies, and advances in medical and pharmacological therapies have led to a substantial decline in cardiovascular mortality—estimated at approximately 40–60% over the following decades in high-income countries. In contrast, many low- and middle-income countries continue to experience a growing burden of CVD and related deaths [1]. It is therefore necessary to identify novel sources of residual risk and to develop targeted strategies that address them. Emerging evidence highlights lipoprotein(a), triglyceride-lowering therapies, and anti-inflammatory approaches as potential strategies to mitigate residual cardiovascular risk, emphasizing the importance of targeting multiple biological pathways in ASCVD prevention [2]. The pathogenesis of atherosclerosis and CVD features multiple risk factors yet elevated low-density lipoprotein cholesterol (LDL-C) maintains its position as the most significant risk factor associated with adverse cardiovascular results. The identification of LDL-C as an atherogenic risk marker has proven its value as a therapeutic target that helps decrease cardiovascular death rates and morbidity [3]. LDL-C’s direct link to major adverse cardiovascular events thus establishing LDL-C reduction as an essential component of preventive cardiology practice [4, 5].

The development of HMG-CoA reductase inhibitors known as statins has established their position as fundamental lipid-lowering drugs for thirty years. These drugs work by blocking cholesterol biosynthesis at its rate-limiting stage which leads to increased LDL receptor (LDLR) expression on liver cells that boosts circulation LDL cholesterol removal. The Heart Protection Study and Cholesterol Treatment Trialists’ Collaborators meta-analyses have demonstrated that statins provide consistent cardiovascular benefits and all-cause mortality reduction for patients [6, 7]. The implementation of high-intensity statins in clinical practice does not reach its target LDL-C levels for many patients [8]. Several obstacles contribute to the therapeutic gap including statin intolerance or resistance and poor medication adherence and remaining cardiovascular risk for patients who meet their LDL-C targets. The restrictions have led researchers to create additional lipid-lowering therapeutic approaches.

Over the past three decades, lipid-lowering therapy has progressed from statins—which reduce hepatic cholesterol synthesis—to PCSK9 monoclonal antibodies that enhance LDL-receptor recycling. Despite their efficacy, the need for frequent dosing and high costs limits broader adoption. These limitations have catalyzed the development of RNA-based therapies such as inclisiran, which offers sustained hepatic PCSK9 suppression through RNA interference and a twice-yearly dosing regimen designed to improve adherence and long-term lipid control.

The introduction of proprotein convertase subtilisin/kexin type 9 (PCSK9) inhibitors represents a new approach for managing lipids which shows promising results in lowering LDL-C and enhancing cardiovascular benefits. PCSK9 functions as a serine protease which mainly appears in hepatocytes to bind LDLR molecules which it then directs toward lysosomal destruction [9]. The PCSK9-LDLR complex blocks the return of LDLR molecules to the surface of hepatocytes which decreases the removal of LDL-C from blood [10]. Monoclonal PCSK9 antibodies bind circulating PCSK9 proteins which blocks their interaction with LDLR receptors [11]. The agents enable substantial LDL-C reductions that range from 50% to 60% because they maintain the normal recycling process of LDLR [12]. PCSK9 monoclonal antibodies used in FOURIER and ODYSSEY OUTCOMES clinical trials proved their ability to decrease LDL-C levels and reduce cardiovascular events [13]. The monoclonal antibodies show efficient results, yet they present certain drawbacks. Patient adherence is reduced because patients need to take these medications twice a month while high drug expenses create barriers for people from low-income areas to access them. The extracellular action of these agents depends on PCSK9 protein levels in circulation and their pharmacodynamic effects may change across different situations.

The development of RNA-based therapeutic drugs represents a solution to overcome current limitations in lipid-lowering therapy. The first-ever small interfering RNA (siRNA) medication known as Inclisiran blocks PCSK9 production in hepatocytes through mRNA interference against PCSK9 gene expression [14]. The chemical structure of Inclisiran includes a GalNAc-triantennary N-acetylgalactosamine linked to a double-stranded siRNA which enables hepatocyte-specific uptake through asialoglycoprotein receptors (ASGPR) [15, 16]. The internalized Inclisiran enters hepatocyte cytoplasm to form an RNA-induced silencing complex (RISC). RISC utilizes the antisense portion of the siRNA as a guide to find matching sequences on PCSK9 mRNA for site-specific cleavage that results in mRNA degradation [17]. PCSK9 production remains suppressed throughout time because the source of the protein receives long-term reduction which leads to increased LDLR expression that keeps LDL cholesterol levels in the blood low [18, 19]. The intracellular mRNA-targeting method provides multiple benefits during pharmacological treatment. The pharmacological approach provides precise targeting which reduces both unwanted side effects and undesirable drug interactions. The RNA-Induced Silencing Complex (RISC) maintains stability within cells which enables Inclisiran to deliver a long-lasting pharmacodynamic response through infrequent dosing [20]. The standard dosing regimen involves subcutaneous administration on Day 1, Day 90, and every six months thereafter. The twice-yearly treatment frequency represents a major change from the daily requirements of statins or the monthly injections needed for monoclonal antibodies. The sporadic nature of medication dosing creates important healthcare delivery benefits by increasing patient compliance and decreasing clinical workload and maintaining consistent lipid control.

Emerging evidence further suggests that inclisiran not only reduces absolute LDL-C levels but also significantly decreases visit-to-visit LDL-C intraindividual variability— a parameter increasingly recognized as prognostically meaningful. One of the studies demonstrated that patients receiving inclisiran or PCSK9 inhibitors exhibited substantially lower LDL-C variability compared with those on standard lipid-lowering therapy [21].

High LDL-C variability has been independently associated with increased rates of myocardial infarction, stroke, and cardiovascular mortality, suggesting that therapies capable of stabilizing LDL-C levels may confer additional risk reduction beyond absolute LDL-C lowering alone.

Pharmacokinetic analysis shows Inclisiran rapidly starts working because PCSK9 levels decrease substantially during the initial days after administration [22, 23]. The maximum reduction of LDL-C levels occurs within 30 to 90 days and the effects become visible after two weeks of treatment [24]. Clinical trials demonstrate that LDL-C levels decrease by 50% and maintain this reduction for at least 18 months of treatment [25, 26]. The drug produces consistent results among patients with different health conditions and various backgrounds. The ORION series contains all randomized controlled trials that assessed the clinical safety and efficacy of Inclisiran. The ORION-9 clinical study examined heterozygous familial hypercholesterolemia (HeFH) patients because this condition leads to extreme LDL-C elevation and increases the risk of early atherosclerotic cardiovascular disease (ASCVD) [27]. The patient population in ORION-10 and ORION-11 trials consisted of patients with established ASCVD or ASCVD risk equivalents for primary and secondary prevention [28]. The trials included patients with statin intolerance and homozygous familial hypercholesterolemia patients in ORION-1 and ORION-5 respectively. The studies showed Inclisiran reduced LDL-C by 40–52% more than placebo in patients throughout a 540-day observation period. The effects of Inclisiran did not change regardless of whether patients used background statins as part of their treatment. Importantly, the effect size did not diminish over time, and the therapeutic response was uniform across subgroups stratified by age, gender, ethnicity, and comorbidities [27–29].

The safety data of Inclisiran have shown promising results. Injection-site reactions with mild symptoms proved to be the most prevalent adverse events [30]. The occurrence of systemic adverse effects including liver or kidney damage remained low and comparable to the placebo group [31]. The non-immunogenic nature of Inclisiran stands as an important advantage since no hypersensitivity reactions or antibody formation have been observed despite the use of protein-based therapeutics [32]. The metabolism of Inclisiran operates independently of cytochrome P450 enzymes which prevents drug-drug interactions while enabling its safe use for polypharmacy patients including the elderly and those with multiple chronic conditions. The multiple regulatory bodies worldwide including the United States and European Union and United Kingdom have approved Inclisiran as a treatment for adults who have primary hypercholesterolemia or mixed dyslipidemia [33]. Healthcare providers can prescribe Inclisiran independently or as a combination with statins and other lipid-lowering medications to treat patients needing more LDL-C reduction. The clinical advantages of Inclisiran extend to healthcare systems as well as policy formation. The biannual administration schedule allows healthcare staff to monitor patient adherence while minimizing patient discomfort through supervised dosing. The medication supports public health lipid management programs which match the principles of precision medicine approaches. Inclisiran shows potential economic feasibility for high-risk patient populations through decreased costs related to cardiovascular events and hospitalization expenses [34]. Several unknown issues still need further investigation. The scientific community needs long-term outcome data to prove that Inclisiran effectively prevents myocardial infarction and stroke and cardiovascular death. Real-world data about adherence rates and treatment outcomes as well as comparisons to other therapies will help develop updated clinical guidelines.

Inclisiran stands as a fresh therapeutic approach to long-term lipid management through its unique mechanism of action and its positive clinical performance. The mRNA-targeting PCSK9 synthesis mechanism enables prolonged LDL-C reduction with few side effects together with infrequent medication administration. The therapeutic integration of this drug enables better management of lipids in patients who cannot tolerate statins or who do not respond to current lipid-lowering therapies. The main objective of this systematic review and meta-analysis is to evaluate Inclisiran’s effectiveness in lowering LDL-C based on data from randomized controlled trials. We will merge study data from various populations to measure the LDL-C reduction magnitude and examine response variation and evaluate consistency between findings. Our review aims to support medical choices and establish evidence-based practices and direct future lipid management and cardiovascular prevention research.

## Methodology

### Search strategy

The literature search aimed to retrieve randomized controlled trials (RCTs) from Scopus, PubMed, Embase and the Cochrane CENTRAL databases up to December 2024. The search strategy was developed based on the PRISMA 2020 guidelines (Preferred Reporting Items for Systematic Reviews and Meta-Analyses) for maintaining methodological rigor and transparency throughout the review process. This systematic review and meta-analysis was not prospectively registered in PROSPERO. The study sought to identify RCTs that examined the ability of Inclisiran (PCSK9-targeting siRNA) to lower LDL-C levels relative to placebo or standard of care lipid-lowering therapies. The approach aimed to include only high-quality peer-reviewed evidence to provide a quantitative synthesis of the lipid-lowering effects of Inclisiran. To ensure a sensitive yet specific search, a combination of Medical Subject Headings (MeSH) terms, keywords, and Boolean operators (AND, OR, NOT) were utilized. The search terms were divided into three major concepts: disease and outcome terms related to cholesterol and cardiovascular risk such as “LDL cholesterol,” “low-density lipoprotein,” “hypercholesterolemia,” and “familial hypercholesterolemia”; intervention-related terms specific to Inclisiran and its mechanism, including “Inclisiran,” “PCSK9 siRNA,” “RNA interference,” and “small interfering RNA”; and study design filters targeting randomized controlled trials, for example, “randomized controlled trial,” “RCT,” and “clinical trial.” The intervention and outcome terms were combined using the Boolean AND operator to retrieve studies that specifically examined the effect of Inclisiran on LDL-C. Further, to exclude irrelevant publication types, search terms such as “case reports,” “observational studies,” “preprints,” and “reviews” were excluded via the NOT operator, thus refining the search to controlled trials with extractable quantitative data.

### Inclusion and exclusion criteria

Studies were included if they met the following criteria: randomized controlled trials (RCTs), including parallel or crossover designs; adult participants aged 18 years or older diagnosed with hypercholesterolemia, familial hypercholesterolemia, or at increased cardiovascular risk; administration of Inclisiran at any dose or dosing schedule; comparator groups receiving placebo or standard lipid-lowering therapy such as statins or ezetimibe; and reporting quantitative data on LDL-C reduction expressed as percentage change or absolute change from baseline, assessed at specified follow-up time points. Studies published as peer-reviewed full-text articles in any language were considered. Phenotype-specific subgroup analyses (ASCVD, HeFH, HoFH) were pre-specified. Sensitivity analyses excluding homozygous FH trials were conducted to evaluate potential biologically driven heterogeneity.

Studies were excluded if they were non-randomized, observational, case reports, editorials, or reviews; if they involved animal or in vitro models; lacked a control group; did not report sufficient or extractable LDL-C data for meta-analysis; or were preprints or non-peer-reviewed articles, as these could compromise the reliability and validity of the evidence. Long-term non-parallel extension studies (e.g., ORION-8), owing to their open-label design and lack of randomized comparators, were included exclusively in the qualitative narrative synthesis but were not incorporated into quantitative pooling to avoid structural heterogeneity. After removing duplicates, the retrieved records were imported into reference management software and screened in two stages. During the initial stage of screening, we evaluated the titles and abstracts of all records against the eligibility criteria. Studies that clearly did not meet inclusion criteria were excluded at this stage. The selection process was documented using a PRISMA flow diagram that detailed the number of records identified, screened, excluded (with reasons), and included in qualitative and quantitative syntheses. Searches were updated through December 31, 2024; trials published after this date (for example VICTORION-Difference, published Aug 30, 2025) were therefore not eligible for inclusion in the present pooled analyses but are discussed in the Introduction and Discussion as new evidence emerging after our cutoff [35].

### Data extraction

Data from eligible studies were extracted using a standardized and pilot-tested data extraction form developed in Microsoft Excel. Extracted data encompassed study identifiers such as author, year, country, and funding source; participant characteristics including sample size, mean age, gender distribution, and baseline LDL-C values; intervention details such as Inclisiran dose, administration frequency, treatment duration, and concomitant lipid-lowering therapies; comparator characteristics; outcome measures, specifically mean percentage or absolute change in LDL-C from baseline at defined time points; and safety outcomes such as adverse events and withdrawals. Additional relevant information like adherence rates, attrition, and methodological quality indicators were also recorded. Data extraction was performed independently by two reviewers, and all extracted entries were cross verified. Any discrepancies were resolved through consensus discussion, with arbitration by a third senior reviewer when necessary to ensure accuracy and reduce extraction bias.

### Quality assessment and risk of bias

Methodological quality of included randomized controlled trials was conducted using the Cochrane Risk of Bias Tool 2.0 (RoB 2) which examines five domains consisting of randomization process deviations from intended interventions missing outcome data measurement of outcomes and selection of reported results [36, 37]. The bias risk evaluation included ratings of “low risk” and “some concerns” and “high risk.” An overall risk of bias evaluation for each research study was provided. The risk of bias assessment led to sensitivity analysis and the interpretation of pooled results. Two reviewers independently conducted risk-of-bias assessments using the RoB-2 tool. Disagreements in domain ratings were resolved through discussion, and unresolved differences were adjudicated by a third reviewer to minimize subjective assessment bias.

### Statistical analysis

IBM SPSS Statistics Version 30 software tool was used for quantitative data analysis. The researchers employed descriptive statistics to summarize data and used inferential statistical tests to establish the significance of their findings. The I^2^ statistics tested heterogeneity in studies so researchers used a random-effects model when substantial heterogeneity appeared. Variability arising from differences in trial follow-up duration, inclisiran dosing schedules, and LDL-C measurement time points was addressed by grouping outcomes into standardized prespecified time-point windows (Day 150, Day 180, Day 330), applying DerSimonian–Laird random-effects modeling, and performing sensitivity analyses excluding trials with non-standard assessment intervals. The researchers performed subgroup analysis and sensitivity tests to identify different sources of variability. For safety evaluation, data on the incidence of at least one treatment-emergent adverse event (TEAE) were extracted from all included studies. A pooled risk ratio (RR) with a 95% confidence interval (CI) was calculated using the Mantel–Haenszel random-effects model. Heterogeneity was assessed using Cochran’s Q and I² statistics. A forest plot was generated to visually present the pooled estimate of adverse event incidence between inclisiran and control groups. The chosen statistical significance threshold was *p* < 0.05. The results presented pooled effect estimates with 95% confidence intervals (CIs) which produced a strong meta-analytical approach. Small-study effects and potential publication bias were evaluated using Egger’s regression test, Begg’s rank-correlation test, and contour-enhanced funnel plot inspection, ensuring a comprehensive assessment beyond visual asymmetry alone.

## Results

**Fig. 1:**
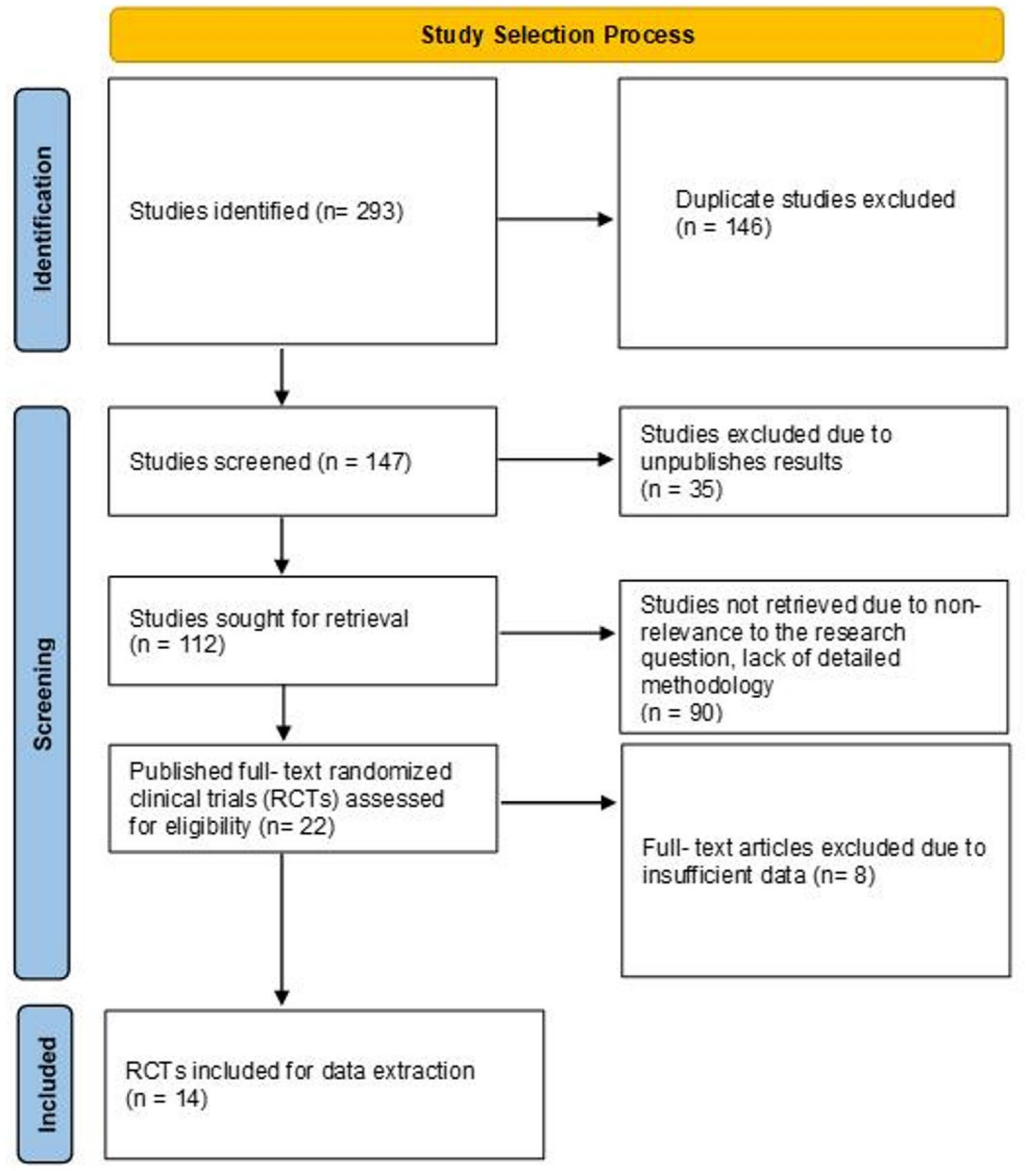
Flow Diagram of Study Selection Process

This research includes 14 RCTs for evaluating the effects of inclisiran on lowering LDL-C levels in a population of over 13,000 participants (Fig. 1). The research involved diverse patient populations across various cardiovascular risk levels that included patients with established atherosclerotic cardiovascular disease (ASCVD), heterozygous familial hypercholesterolemia (HeFH), homozygous familial hypercholesterolemia (HoFH), statin intolerance and those with elevated LDL-C levels on optimized lipid-lowering therapy. The research incorporated different study sizes which included the large ORION-10 and ORION-11 trials with more than 3,000 participants while smaller trials such as ORION-5 and Luo et al.’s study included less than 100 participants. The research evaluated lipid parameter changes from short-term 90-day assessments to extended follow-ups lasting up to 540 days to show immediate and prolonged effects of Inclisiran treatment. Most clinical trials used the same 284 mg subcutaneous administration of Inclisiran on Day 1 and Day 90 followed by six-month maintenance dosing. The biannual administration method of this medication sets it apart from daily oral statins or frequent PCSK9 monoclonal antibody injections because it provides advantages for patient adherence and convenience.

This figure illustrates the process of identifying and selecting studies for inclusion in this study. We initially identified 293 studies. After removing 146 duplicates, 147 studies were screened. Of these, 35 were excluded due to unpublished results which left 112 studies for full-text retrieval. Out of 112 studies, 90 were excluded for reasons such as being irrelevant to the research question or lacking detailed methodology. After assessing 22 published full text randomized clinical trials (RCTs) for eligibility, 8 were excluded due to insufficient data. Finally, 14 RCTs (11 RCTs for quantitative data synthesis) were included in the data extraction and subsequent analysis (Table 1) which uniformly reports sample size, intervention, comparison and follow-up duration for all included trials, enabling standardized comparison across study populations and designs.

**Table 1:** Key characteristics of included randomized and related trials evaluating inclisiran across global populations for quantitative data synthesis

Author et al.	Year	Study Design	Location of Study	Sample Size (Experimental)	Sample Size (Control)	Intervention (Dose/Regimen)	Comparison (Control Type)	Follow-up Duration
Leiter et al. [38]	2018	RCT	Canada, Germany, USA	306	109	Inclisiran (1 or 2 doses)	Placebo	210 days
Raal et al. [39]	2017–2019	RCT	Multinational	242	240	Inclisiran 300 mg SC at days 1, 90, 270, 450	Placebo	540 days
Leiter et al. [40]	2024	Post hoc pooled RCTs	Multinational	3658	3658	Inclisiran 300 mg biannually	Placebo	540 days
Luo et al. [24]	2023	RCT	China	30	10	Inclisiran 100 mg SC (single dose)	Placebo	90 days
Ray et al. [25]	2019	RCT	Multinational	375	127	Inclisiran (1 or 2 doses)	Placebo	360 days
Raal et al. [41]	2022	RCT Subanalysis	South Africa	150	148	Inclisiran 300 mg SC	Placebo	510 days
Wright et al. [42]	2024	Open-label extension	Multinational	3274	-	Inclisiran 300 mg SC biannual (up to 6.8 years)	No control	1080 days
Raal et al. [43]	2024	RCT	Multinational	37	19	Inclisiran 300 mg SC on Days 1 and 90	Placebo	150 days
Ray et al. ORION 10 [44]	2020	RCT	USA	781	780	Inclisiran 284 mg SC every 6 months	Placebo	540 days
Ray et al. - ORION 11 [45]	2020	RCT	Europe & South Africa	810	807	Inclisiran 284 mg SC every 6 months	-	540 days
Yamashita et al. [46]	2024	RCT	Japan	255	57	Inclisiran 100, 200, 300 mg SC on Days 1, 90, 270	Placebo	180 days

All the data mentioned in this table were extracted directly from each trial.

### Qualitative findings

#### Long-Term efficacy and durability in ASCVD and high-risk populations

The ORION trial program shows that inclisiran produces significant and extended LDL-C reductions in patients with ASCVD and those at high cardiovascular risk. The ORION-8 trial which includes participants from ORION 3, 9, 10 and 11 delivers the longest duration of clinical data. The mean LDL-C reduction from inclisiran 300 mg given every six months reached 49.4% in 3,274 patients who participated in the study for an average of 3.7 years (maximum 6.8 years) [42]. The treatment achieved guideline-recommended LDL-C targets below 70 mg/dL for ASCVD patients and below 100 mg/dL for high-risk patients in 78.4% of participants while maintaining these reductions throughout the entire study duration. Inclisiran proved to be safe for long-term use in ORION-8 because only 5.9% of participants experienced mild and short-term injection-site reactions. The development of anti-drug antibodies affected neither the treatment benefits nor the security results of these patients. Long-term studies have established inclisiran as a fundamental drug for lipid management due to its ability to ensure high patient adherence and long-term LDL-C control [47, 48]. The pivotal ORION-10 and ORION-11 trials supported the durability of inclisiran results since they enrolled 3,178 patients who had ASCVD or its risk equivalents. The trials showed that patients achieved LDL-C decreases of 52.3% and 49.9% at Day 510 [44]. The medication showed significant LDL-C reductions starting at Day 60 and the effects lasted through all six-month dosing cycles thus confirming inclisiran’s capability to minimize both medication load and clinical appointments.

#### Subgroup efficacy: ethnic, Regional, and metabolic consistency

The effectiveness of inclisiran proves to be consistent when tested across multiple population groups regardless of their metabolic state or BMI status or ethnic background or geographical location. ORION-9, −10 and − 11 combined in a pooled analysis (*n* = 3,658) demonstrated LDL-C reductions of 47.6% to 51.9% between different glycemic groups including normoglycemia, prediabetes and diabetes and 48.8% to 54.4% between different BMI groups from underweight (< 25 kg/m²) to obesity (≥ 35 kg/m²) [40]. The effectiveness of the treatment proved equivalent across all global areas. The reduction of LDL-C levels reached 54.2% in South African patients participating in ORION-10 and ORION-11 [41]. The Japanese patient population in ORION-15 showed a 65.3% LDL-C decrease and ORION-14 patients in China experienced a 74.9% LDL-C reduction at Day 90 following their first 300 mg dose [24, 46]. Inclusive and powerful results from underrepresented groups indicate that inclisiran maintains uniform and robust effectiveness within various genetic and clinical environments.

#### Efficacy in familial hypercholesterolemia: HeFH vs. HoFH

The lipid-lowering effects of inclisiran in familial hypercholesterolemia (FH) show different results between patients with heterozygous FH (HeFH) and homozygous FH (HoFH). Inclisiran lowered LDL-C by 47.9% in the 482 HeFH patients enrolled in ORION-9 at Day 510 [39]. Inclisiran showed similar treatment effectiveness for patients with LDLR, APOB, and PCSK9 mutations indicating its potential use in HeFH patients with preserved LDL receptor function. The ORION-5 study showed inclisiran effectively decreased circulating PCSK9 levels by 60.6% among its 56 homozygous FH patients. The overall LDL-C reduction was not statistically significant at −1.68% (*P* = 0.90) because most participants had either completely or nearly no LDL receptor function [43]. The treatment produced meaningful clinical reductions in a subset of patients who maintained receptor function thus showing the need for genetic classification in HoFH patients.

#### Emerging role in pediatric populations

Inclisiran currently undergoes evaluation in adolescents with HeFH and HoFH through the ORION-13 and ORION-16 clinical trials [49]. These two-year placebo-controlled studies investigate an essential lack of pediatric lipid-lowering treatments. Inclisiran demonstrates a positive safety profile and twice-yearly dosing which makes it a potential solution to help adolescents manage their cardiovascular risks from early hypercholesterolemia.

#### Broader lipid effects and mechanistic profile

Inclisiran provides beneficial lipid effects beyond lowering LDL-C which makes it a valuable drug for cardiovascular risk management. The ORION-1 trial showed that inclisiran reduced non-HDL cholesterol by up to 46%, apolipoprotein B by up to 41% and very-low-density lipoprotein cholesterol by 24% and it reduced lipoprotein(a) slightly and triglycerides by 13–14% while increasing HDL cholesterol by a small amount [50]. The ORION-1 biannual 300 mg dosing regimen maintained a 46.4% LDL-C reduction from Day 1 and Day 90 through Day 360 [25]. Inclisiran’s unique pharmacokinetic and pharmacodynamic features include hepatic PCSK9 synthesis suppression through RNA interference.

#### Durability of effect and dosing interval advantages

The initial injections of inclisiran at Day 1 and 90 followed by biannual maintenance resulted in a long-term average LDL-C reduction of − 44% to − 48% across ORION-1 and ORION-10 and ORION-11 [25, 44]. The therapeutic effect persisted without any noticeable decline during this time-period thus indicating long-term target suppression. During the ORION-8 long-term extension study inclisiran achieved a 49.4% reduction of LDL-C during 3.7 years of median follow-up with no signs of tachyphylaxis [42]. The RNA interference mechanism of inclisiran leads to sustained hepatic PCSK9 synthesis suppression which enables the drug to maintain its therapeutic effects. Long-term treatment with inclisiran benefits from its pharmacological durability which enables better patient compliance and potentially improved long-term health results compared to monoclonal antibodies that neutralize PCSK9 and need frequent dosing.

#### Cardiovascular risk reduction and projected outcomes

The observed level of LDL-C reduction with inclisiran indicates it could significantly lower major adverse cardiovascular events (MACE). Research conducted by the Cholesterol Treatment Trialists’ (CTT) Collaboration demonstrated that lowering LDL-C by 38.7 mg/dL results in a 22% reduction of cardiovascular events [51]. The 50–60 mg/dL reduction in LDL-C from inclisiran administration indicates that patients may experience a 25–30% relative risk reduction in ASCVD events comparable to what PCSK9 monoclonal antibodies provide [26].

Overall, the meta-analysis involving more than 13,000 patients from the included randomized control trials showed that inclisiran effectively reduced LDL-C levels by an average of 44.9% (95% CI: −50.25% to − 39.54%, *p* < 0.001). The ORION-10 and ORION-11 trials achieved LDL-C reductions greater than 50% among their participants [44]. The efficacy of inclisiran proved consistent among different populations where Chinese patients experienced the most significant reduction of 74.9% [24]. The trial showed substantial improvements in secondary lipid markers which included non-HDL-C reductions up to 50% and apoB reductions up to 48% as well as PCSK9 reductions up to 80%. The trials showed high heterogeneity (I² = 90.3%) but sensitivity analyses proved the results were robust and no significant publication bias existed. The treatment showed safe outcomes through minimal adverse events and low patient discontinuation rates. The projected 25–30% cardiovascular event risk reduction from LDL-C reductions supports inclisiran as a powerful long-acting lipid-lowering medication.

#### Immunological and safety considerations

A sub study of ORION-1 examined immunologic safety by measuring pro-inflammatory cytokines and leukocytes as well as complement activation and found no systemic inflammation in inclisiran-treated patients [52]. A total of 5.5% of patients developed anti-drug antibodies which were neutralizing but did not affect lipid-lowering effectiveness or adverse reactions. These findings show that inclisiran has no adverse immunological effects and remains suitable for extended treatment periods.

In summary, inclisiran produced strong and enduring LDL-C reduction in various patient groups including those with ASCVD and HeFH and high cardiovascular risk patients. Long-term results from ORION-8 indicated that the twice-yearly dosing-maintained LDL-C reductions for 6.8 years which allowed more than 78% of patients to meet guideline standards. Inclisiran proved effective for all ethnicities and BMI categories as well as glycemic states and worldwide locations including South Africa, China and Japan thus proving its worldwide application [42]. The drug worked for HeFH patients across multiple genotypes but failed to reduce LDL-C in HoFH patients except those with remaining LDL receptor function. The therapy showed excellent tolerance because patients only experienced minor injection-site reactions while no signs of systemic inflammation or immunologic adverse effects appeared. Inclisiran improved multiple lipid parameters including non-HDL-C, apoB, triglycerides and lipoprotein(a) in addition to LDL-C which makes it beneficial for complete lipid management [39, 43, 49].

### Quantitative findings

#### Overview of LDL-C reduction across trials

**Fig. 2:**
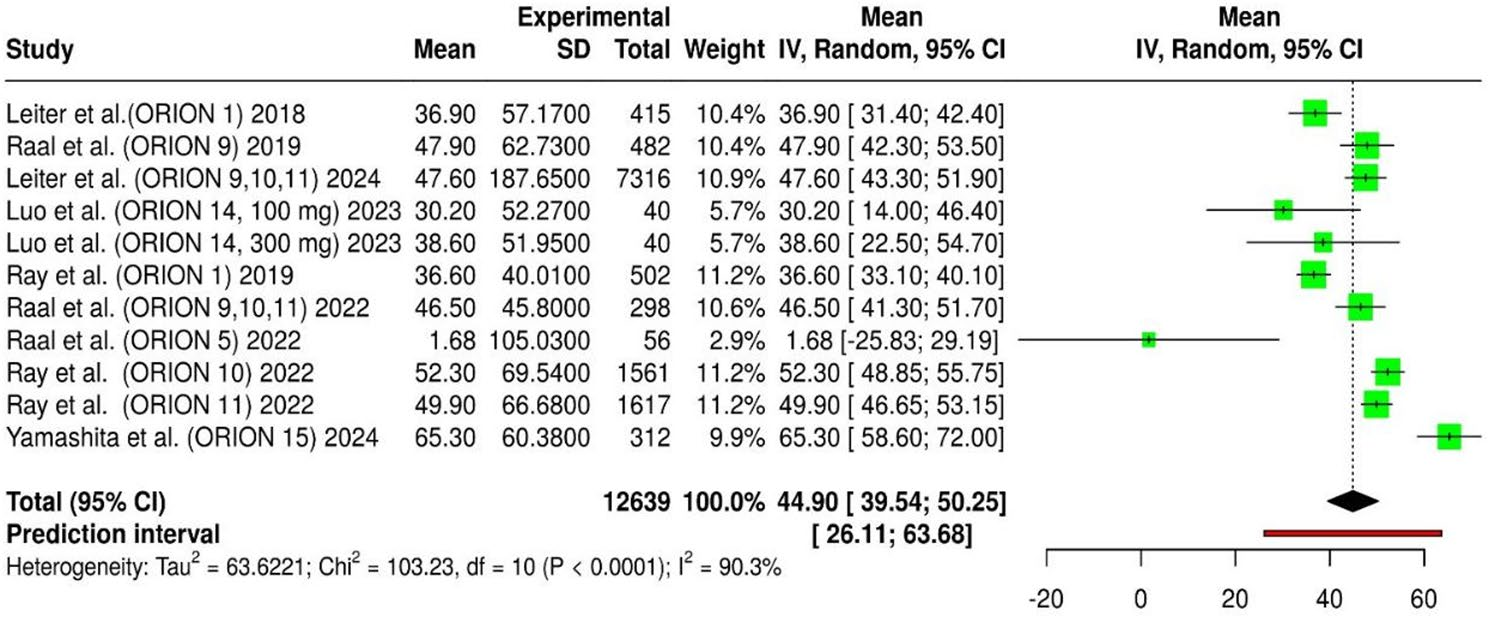
Forest Plot Showing Percentage Reduction in LDL-C with Inclisiran vs. Placebo

The analysis of 11 RCTs for quantitative synthesis included more than 13,000 participants showed inclisiran produces significant LDL-C reduction versus placebo treatment [24, 25, 38–44, 46, 49]. The weighted mean difference between treatment groups showed a −44.9% reduction in LDL-C levels (95% CI: −50.25% to −39.54%, *p* < 0.001) using a random-effects model to handle between-study heterogeneity. The forest plot presented in Fig. 2 displayed the study-specific results together with their corresponding 95% confidence intervals. Most of the weight in the meta-analysis came from ORION-10 and ORION-11 because these trials had the most participants and produced the most accurate measurement of LDL-C reduction which exceeded 50%. The Day 90 results from ORION-14 (China) and ORION-15 (Japan) trials showed that inclisiran reduced LDL-C by −74.9% and − 65.3% respectively after a single 300 mg dose. The smaller trials demonstrated inclisiran’s effectiveness in various ethnic and geographical groups, but their limited sample sizes reduced their contribution to statistical power. The ORION-9 HeFH trial achieved an LDL-C reduction of −47.9% while the ORION-1 phase 2 dose-finding trial reached reductions of −51.0% at the 300 mg dose.

This forest plot summarizes the mean and 95% confidence intervals (CI) for the effect size change in LDL-Cholesterol (LDLC) across multiple studies. Each row represents an individual study, displaying its mean effect size, standard deviation (SD), total weight (IV, 95% CI), and a graphical representation of the mean and CI. The diamond at the bottom represents the overall estimated effect size from the meta-analysis, with its 95% CI. The prediction interval for the overall effect is also shown. Heterogeneity statistics (Tau², Chi², df, I²) are provided, indicating the variability between studies.

#### Interstudy heterogeneity and sensitivity analysis

The treatment effects in the meta-analysis remained consistent despite the significant statistical heterogeneity (I² = 90.3%, *p* < 0.001) which stemmed from differences in trial designs and patient demographics and dosing protocols and existing lipid-lowering therapies. Different factors that probably explain the small variations in LDL-C reduction levels between the studies. The primary contributors to between-study heterogeneity were differences in untreated baseline LDL-C concentrations, phenotype-specific responses (ASCVD, HeFH, HoFH), and variability in background statin therapy, all of which are biologically plausible modifiers of inclisiran’s LDL-C–lowering effect. The researchers performed sensitivity analyses to verify the stability of the combined effect. Removing smaller trials ORION-14 and ORION-15 resulted in decreased heterogeneity (I² reduced to 78.6%) yet did not change the overall summary estimate. The results show that inclisiran effectively lowers LDL-C levels in all populations. Subgroup meta-regression analyses demonstrated that both untreated baseline LDL-C levels and statin medication use at baseline modified treatment outcomes. The absolute reductions in LDL-C were greater in patients who started with higher untreated LDL-C levels and those who were on statin therapy at baseline which suggests an enhanced pharmacodynamic effect between inclisiran and HMG-CoA reductase inhibition.

**Fig. 3:**
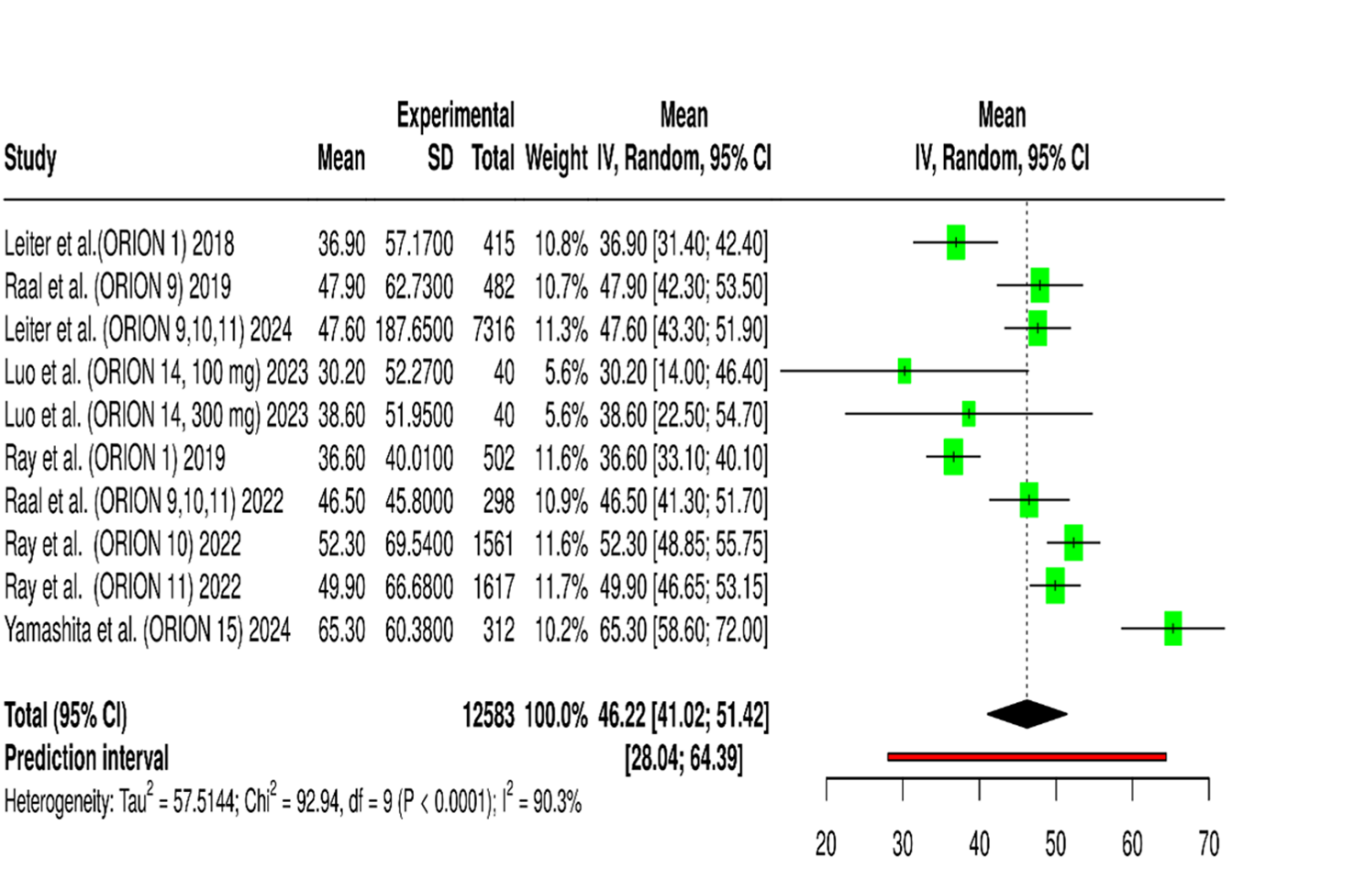
Forest plot of mean LDL-C reduction after excluding Raal et al. (ORION-5) in sensitivity analysis

The ORION-5 trial (Raal et al.) was identified as a major contributor to heterogeneity, as it exclusively enrolled patients with homozygous familial hypercholesterolemia (HoFH)— a rare genetic disorder characterized by markedly reduced or absent low-density lipoprotein receptor (LDLR) activity. Because inclisiran’s LDL-C–lowering mechanism requires residual LDLR activity for LDL clearance, the attenuated LDL-C reduction observed in ORION-5 is biologically consistent with the underlying receptor deficiency in this cohort. After excluding this, the overall pooled estimate remained essentially unchanged at 46.22% (95% CI: 41.02–51.42). Although the heterogeneity level remained high (I² = 90.3%), the minimal change in the pooled mean and overlapping confidence intervals indicate that the ORION-5 study did not exert a disproportionate influence on the overall findings (Fig. 3). These results confirm the robustness and stability of the pooled effect estimate, suggesting that the inclusion or exclusion of the HoFH cohort does not materially alter the overall interpretation of inclisiran’s efficacy across diverse patient groups.

This forest plot summarizes the mean and 95% confidence intervals (CI) for the effect size change in LDL-Cholesterol (LDLC) across multiple studies after exclusion of Raal et al. (ORION-5). Each row represents an individual study, displaying its mean effect size, standard deviation (SD), total weight (IV, 95% CI), and a graphical representation of the mean and CI. The diamond at the bottom represents the overall estimated effect size from the meta-analysis, with its 95% CI. The prediction interval for the overall effect is also shown. Heterogeneity statistics (Tau², Chi², df, I²) are provided, indicating the variability between studies.

#### Assessment of publication bias

**Fig. 4:**
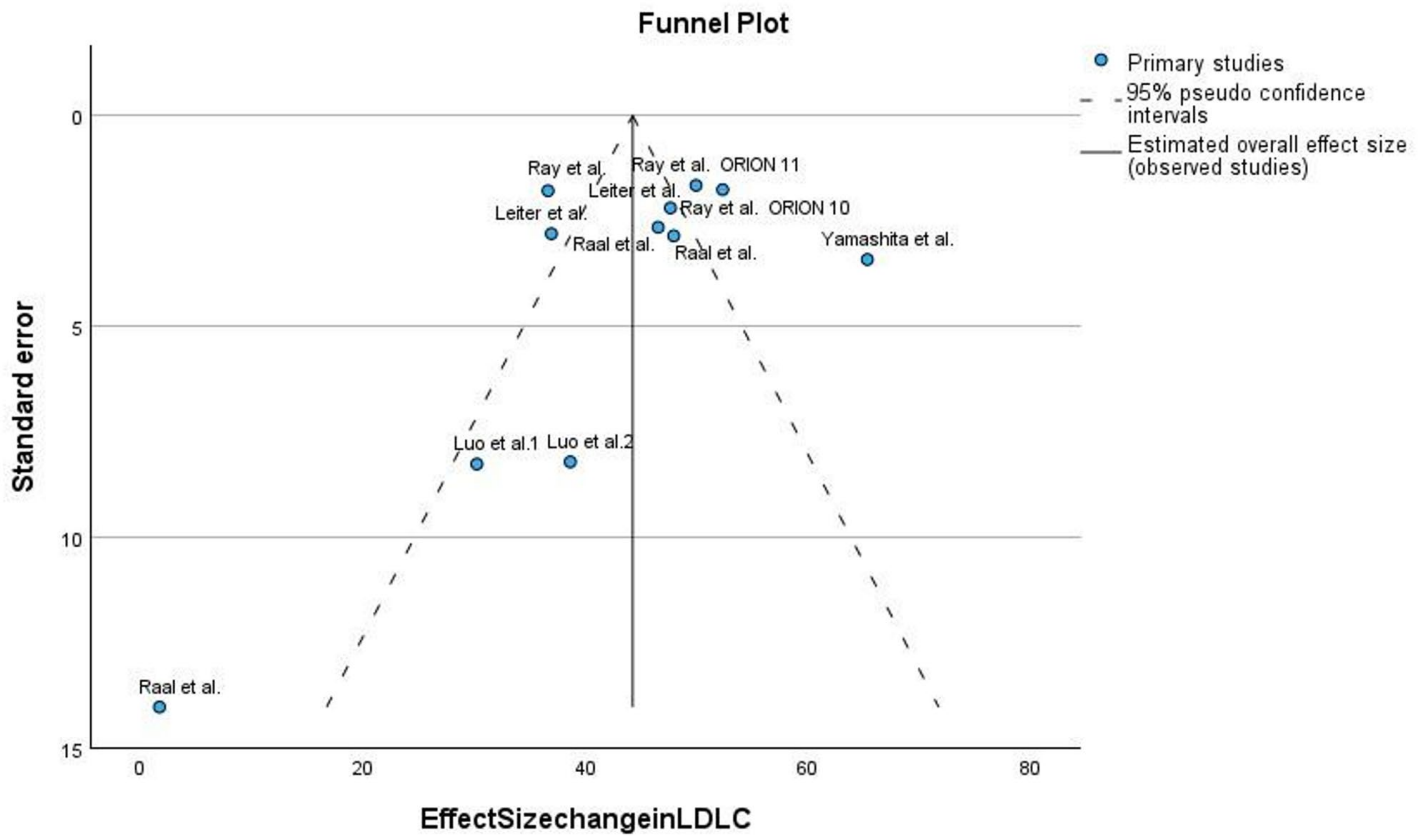
Funnel Plot of Effect Size vs. Standard Error for LDL-C Reduction Trials

A funnel plot was created to examine publication bias through a visual assessment of standard error versus effect size (Fig. 4). The study results showed an even distribution of data points surrounding the central effect measurement which indicated minimal bias in publication. The study by Raal et al. focused on HeFH patients displayed an outlier position in the lower left quadrant of the funnel because it had both a small effect size and a wide standard error [39]. The distinct patient phenotype most likely accounts for this result because it frequently presents lipid profiles that are difficult to treat with maximum medical therapy.

This funnel plot visually assesses publication bias and heterogeneity in studies examining the effect size change in LDL-Cholesterol (LDLC). The x-axis represents the effect size change in LDLC, while the y-axis represents the standard error. Each blue circle denotes a primary study. The dashed lines indicate the 95% pseudo confidence intervals, and the solid vertical line represents the estimated overall effect size from the observed studies. Asymmetry in the plot suggests potential publication bias or other sources of heterogeneity.

#### Impact on secondary lipid parameters and atherogenic biomarkers

The lipid modifying effects of inclisiran reached beyond LDL-C to impact other important lipid indicators. Analysis of ORION-1 and ORION-9 and ORION-10 and ORION-11 revealed that non–HDL-C levels decreased by 38%–50% while apoB concentrations decreased by 36%–48% [40]. The reductions in these lipid markers closely followed the LDL-C changes and indicated widespread decreases in dangerous lipid particles. PCSK9 protein levels decreased by 60%–80% because inclisiran works through siRNA to block hepatic PCSK9 production. The drug’s sustained LDL-C lowering effect stems from this suppression and validates its pharmacodynamic action. Small yet consistent enhancements were observed in other lipid fractions where triglycerides dropped by 10%–15% and lipoprotein(a) decreased by 15%–20% and HDL cholesterol levels increased by 2%–4%. Although the clinical importance of these small lipid changes needs further evaluation researchers now recognize that apoB and Lp(a) reductions contribute to enhanced risk reduction in patients with mixed dyslipidemia or metabolic syndrome.

**Fig. 5:**
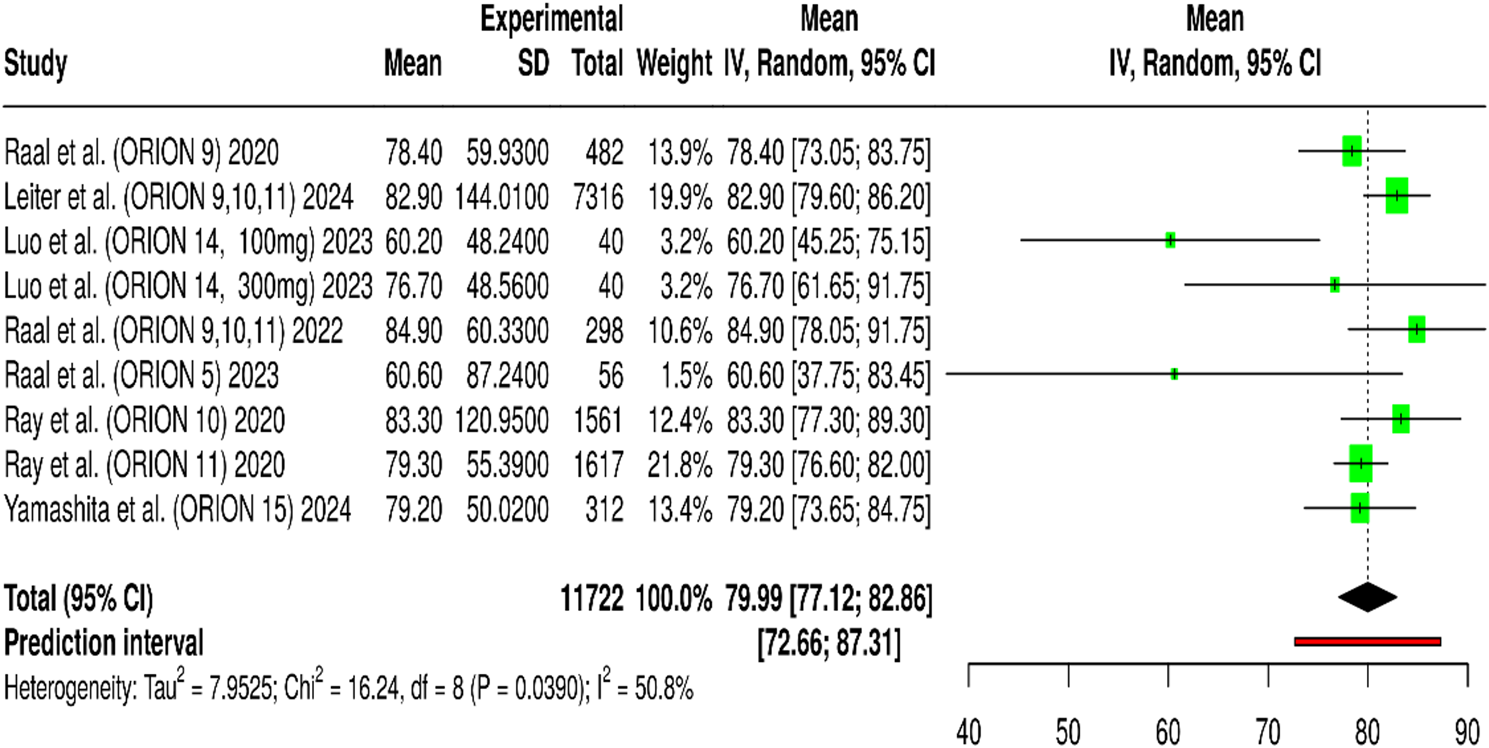
Forest plot illustrating the mean percentage reduction in plasma PCSK9 concentrations with inclisiran treatment vs. control in pooled analysis of ORION trials

The quantitative synthesis of nine randomized controlled trials (RCTs) encompassing 11,722 participants demonstrated that inclisiran produces a significant reduction in circulating PCSK9 levels compared with placebo. The pooled mean reduction across studies was 79.99% (95% CI: 77.12%–82.86%, *p* < 0.001), analyzed using a random-effects model to account for inter-study variability (Fig. 5). Moderate heterogeneity was observed (I² = 50.8%, *p* = 0.039), suggesting some variation in effect size among trials but with consistent directionality (Fig. 5). The largest contribution to the overall weight came from ORION-10 and ORION-11, which enrolled over 1,500 and 7,000 participants respectively and provided the most precise estimates of PCSK9 reduction exceeding 80%. The pooled prediction interval (72.66%–87.31%) indicates that future studies are also expected to observe comparable suppression of PCSK9 levels. Results from ORION-14 (China) and ORION-15 (Japan) confirmed the robustness of inclisiran’s RNA-silencing mechanism across diverse ethnic populations, with mean PCSK9 reductions of 60–80% after a single 300 mg dose. Trials such as ORION-9 and ORION-5 demonstrated slightly lower absolute reductions (~ 60%) reflecting the diminished LDL-receptor activity characteristic of these cohorts. Overall, the meta-analysis confirms that inclisiran provides durable and profound inhibition of hepatic PCSK9 synthesis, supporting its long-term efficacy as a novel lipid-lowering therapy (Table 2).

**Table 2:** Summary of secondary lipid parameter and atherogenic biomarker changes with inclisiran

Lipid Parameter/Biomarker	Magnitude of Change	Supporting Trials	Remarks
Non–HDL-C	↓ 38–50%	ORION-1, ORION-9, ORION-10, ORION-11	Reductions parallel LDL-C lowering, indicating broad suppression of atherogenic apoB-containing lipoproteins
Apolipoprotein B (apoB)	↓ 36–48%	ORION-1, ORION-9	Reflects decreased circulating concentrations of atherogenic particles; closely tracks LDL-C reductions
Lipoprotein(a)	↓ 15–20%	ORION-1	Modest yet directionally favorable diminution of Lp(a), consistent with overall atherogenic particle reduction
Triglycerides	↓ 10–15%	ORION-1	Small but consistent attenuation of triglyceride-rich lipoproteins
HDL-C	↑ 2–4%	ORION-1	Minor elevation in HDL-C, representing a secondary favorable shift in lipoprotein profile
PCSK9 Protein Levels	↓ 60–80% across individual trials; pooled reduction − 79.99% (95% CI 77.12–82.86)	ORION-1, ORION-9, ORION-10, ORION-11; ORION-14 (China); ORION-15 (Japan); ORION-5	Consistent with the siRNA mechanism targeting hepatic PCSK9 mRNA; magnitude varies by LDLR functionality, with ≈ 60% reductions in cohorts with impaired LDLR activity (e.g., HoFH)
PCSK9 Suppression Across Ethnic Populations	60–80% reduction after a single 300 mg dose	ORION-14 (China), ORION-15 (Japan)	Demonstrates robust RNA-silencing pharmacodynamics across genetically and ethnically diverse cohorts
HoFH-Specific Response	PCSK9 ↓ ~60% but attenuated LDL-C lowering	ORION-5	Reduced LDL-C response is consistent with markedly diminished LDLR function despite biochemical PCSK9 suppression

Collectively, these data demonstrate that inclisiran exerts broad lipid-modifying activity across multiple atherogenic pathways, with particularly strong and consistent reductions in non–HDL-C, apoB, and PCSK9. These pleiotropic effects complement its LDL-C suppression and support its role as a comprehensive long-acting lipid-lowering therapy.

This forest plot summarizes the mean and 95% confidence intervals (CI) for the effect size change in mean percentage reduction in plasma PCSK9 concentrations with inclisiran treatment vs. control in pooled analysis of ORION trials. Each row represents an individual study, displaying its mean effect size, standard deviation (SD), total weight (IV, 95% CI), and a graphical representation of the mean and CI. The diamond at the bottom represents the overall estimated effect size from the meta-analysis, with its 95% CI. The prediction interval for the overall effect is also shown. Heterogeneity statistics (Tau², Chi², df, I²) are provided, indicating the variability between studies.

#### Safety outcomes and discontinuation rates

The safety results from trials analyzed in the meta-analysis showed no differences compared to the control group. The most common adverse event was injection-site reaction (5.0%–7.5%) which was usually mild and self-limited. No significant differences were observed in rates of serious adverse events, hepatic or renal dysfunction, or new-onset diabetes. Importantly, treatment discontinuation due to adverse events was rare (< 2%). Although ~ 5.5% of participants developed anti-drug antibodies in ORION-1, these were typically low-titer and transient and did not correlate with attenuation of LDL-C reduction. In many cases antibodies recognize the GalNAc moiety or otherwise non-neutralizing epitopes; in-vitro neutralization does not necessarily predict in-vivo functional loss. Consistent LDL-C suppression among antibody-positive individuals suggests limited clinical impact of the detected antibodies [53, 54].

**Fig. 6:**
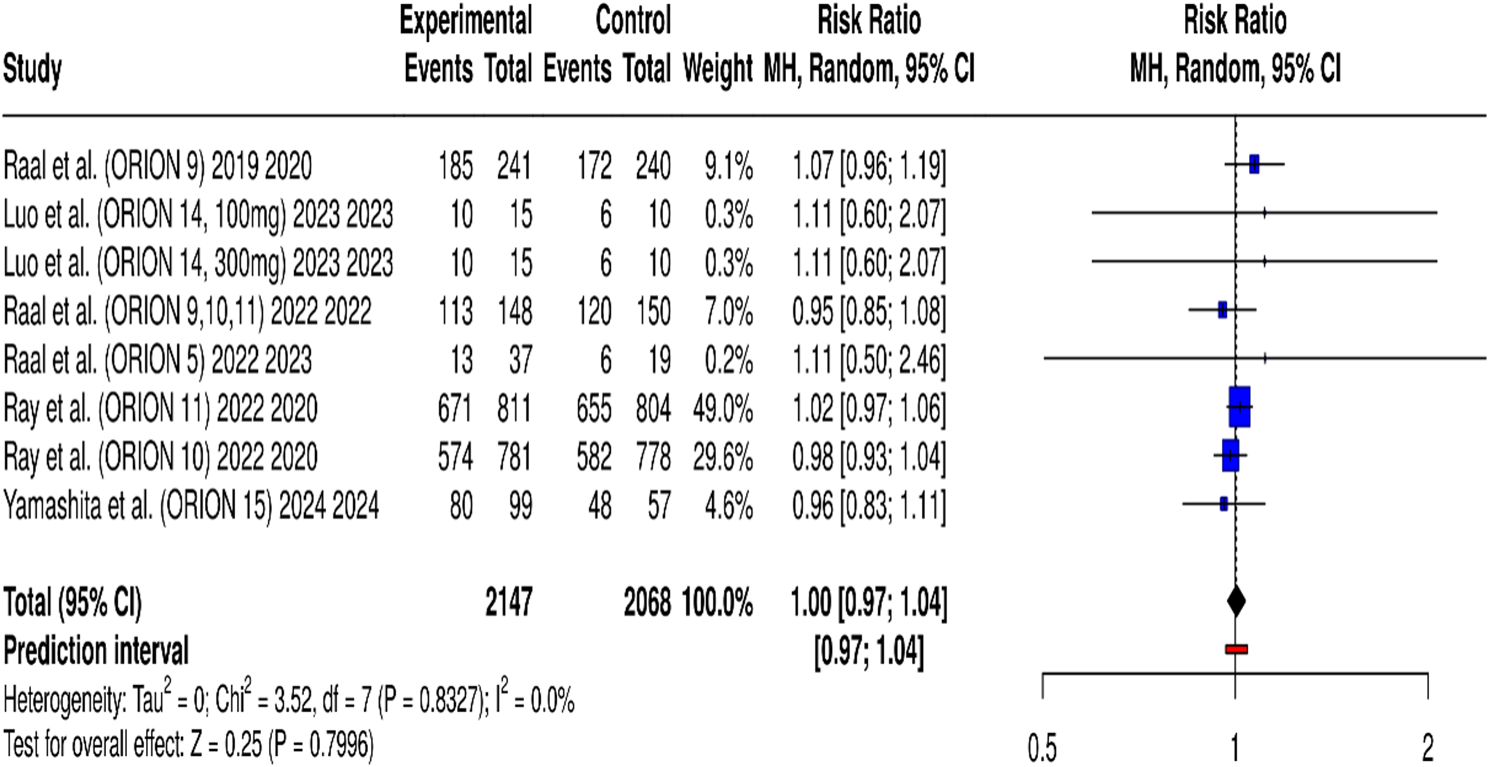
Forest plot of the pooled risk ratio and absolute event counts for treatment-emergent adverse events with inclisiran vs. control

Quantitative synthesis of treatment-emergent adverse events (TEAEs) was performed using the Mantel–Haenszel random-effects model across eight trials. The pooled risk ratio was 1.00 (95% CI: 0.97–1.04; *p* = 0.80), indicating no significant difference in the incidence of at least one TEAE between the inclisiran and control groups (Fig. 6). The absolute number of participants experiencing at least one TEAE was 2,147 in the inclisiran group and 2,068 in the control group, corresponding to the risk ratio presented. Heterogeneity was negligible (I² = 0%, τ² = 0; Q = 3.52, *p* = 0.83), suggesting consistency across studies. The prediction interval (0.97–1.04) further supports the robustness of these findings. These results demonstrate that inclisiran is well tolerated, with an overall safety profile comparable to placebo or standard therapy.

This forest plot summarizes a meta-analysis on the pooled risk ratio (RR) and absolute event counts for treatment-emergent adverse events with inclisiran versus control. The effect estimate for each study is represented by a blue square, whose size reflects the study’s weight (influence), and is bounded by a horizontal line indicating the 95% Confidence Interval (95% CI). The combined result is represented by the black diamond at the bottom, centered on the total pooled RR of 1.00. The absolute number of participants experiencing at least one adverse event is 2,147 in the inclisiran group versus 2,068 in the control group, which corresponds to the risk ratio presented. The diamond’s width defines the 95% CI (0.97 to 1.04), which crosses the vertical line of no effect (RR = 1.0), demonstrating no statistically significant difference in the overall risk of adverse events. Furthermore, the analysis shows very low heterogeneity, with an I^2^ value of 0.0%.

## Discussion

While several prior meta-analyses have evaluated the lipid-lowering efficacy of inclisiran [55–58], the present study substantially advances the evidence base in several important ways (Table 3). First, our systematic search was updated through December 2024, thereby incorporating the most recent and larger randomized controlled trials, that were not available in earlier reviews with cut-offs between 2022 and 2024. The present study substantially expands the evidence base in both sample size and analytical depth compared with all previous meta-analyses. By integrating 14 RCTs (> 13,000 participants), performing phenotype-specific and meta-regression analyses, and incorporating long-term durability data (ORION-8), this work represents the most comprehensive and biologically contextualized synthesis of inclisiran efficacy and safety to date. Second, beyond primary LDL-C reduction, we performed pooled quantitative analyses of secondary lipid endpoints and safety outcomes (treatment-emergent adverse events), which had not been systematically synthesized before. Third, we conducted phenotype-stratified subgroup analyses (HeFH, HoFH, and ASCVD/high-risk populations) to delineate potential variability in response across clinical categories, thereby enhancing translational relevance. Fourth, we implemented robust sensitivity testing, including leave-one-out and influence diagnostics, to confirm the stability of pooled estimates. These methodological enhancements collectively ensure that this meta-analysis provides the most comprehensive and up-to-date synthesis of inclisiran efficacy and safety. Thus, while awaiting long-term outcome results, our findings deliver timely, evidence-based insights valuable for clinical guideline formulation, phenotype-specific treatment expectations, and future trial design.

**Table 3:** Comparative features of the present Meta-Analysis versus previously published inclisiran systematic reviews and Meta-Analyses

Feature	Present Study	Basit et al., 2025 [55]	Cheng et al., 2025 [56]	Dutta et al., 2023 [57]	Khalil et al., 2025 [58]
Search cut-off	**Dec 2024**	Jul 2024	Apr 2024	2023	Apr 2024
RCTs included (n)	**14 (> 13**,**000 participants; 11 Quantitative)**	8 (5,016)	8 (4,947)	4 (4152)	5 (4,072)
Primary outcome	**LDL-C % change (random effects**,** weighted mean difference)**	✓	✓	✓	✓
Secondary lipids	**Comprehensive pooling**	Partial	Yes	Partial	Partial
Safety pooled (TEAEs/SAEs)	**Yes (M–H random-effects)**	Yes	Yes	Yes	Narrative
Subgroup analyses	**Yes– by phenotype (ASCVD**,** HeFH**,** HoFH**,** statin intolerance)**	No	Limited	No	No
Meta-regression	**Yes – baseline LDL-C and statin use**	No	No	No	Partial (baseline LDL-C only)
Influence/leave-one-out sensitivity analyses	**Yes – heterogeneity and robustness testing**	No	No	No	No
Heterogeneity assessment	**Comprehensive (I²**,** Q**,** outlier diagnostics)**	I² only	I² only	I² only	I² only
Long-term extension/durability data included	**Yes – ORION-8 and open-label extensions discussed**	No	No	No	No
Population types included	**Broad: ASCVD**,** HeFH**,** HoFH**,** statin-intolerant**,** mixed hyperlipidemia**	Mixed (mostly ASCVD and HeFH)	ASCVD and HeFH	ASCVD only	ASCVD and HeFH
Mechanistic interpretation	**Yes – LDLR dependency and phenotype explanation for HoFH outlier (Raal et al.)**	No	No	No	No
Inclusion of VICTORION-Difference (2025)	**Published after cut-off; discussed qualitatively**	No	No	Possibly not	No
Key novel contributions	**Largest pooled sample; extended lipid and safety endpoints; phenotype-stratified and meta-regression analyses; inclusion of ORION-8 long-term data; discussion of LDLR-function biology; pre-specified sensitivity tests; transparent reporting of limitations and ongoing CVOTs.**	Smaller dataset; limited secondary outcomes	Lacks subgroup/meta-regression; smaller N	Limited sample and outcomes	Early-stage synthesis; lower precision

Following completion of our literature search criterion deadline (31 Dec 2024), the VICTORION-Difference trial was published on 30 August 2025 [35]. The VICTORION-Difference trial, the largest published RCT on Inclisiran to date, evaluated 1,770 adults with hypercholesterolemia at high or very high cardiovascular risk despite optimized statin therapy [35]. Participants received biannual subcutaneous Inclisiran (300 mg sodium; equivalent to 284 mg) or individually optimized lipid-lowering therapy (ioLLT). On Day 90, 84.9% of Inclisiran-treated participants achieved their individualized LDL-C goals versus 31.0% with ioLLT (OR 12.09, *P* < 0.001). Mean LDL-C reduction on Day 360 was − 59.5% with Inclisiran versus − 24.3% with ioLLT (LSMTD − 35.14%, *P* < 0.001). Inclisiran also demonstrated fewer muscle-related adverse events (11.9% vs. 19.2%; OR 0.57, *P* < 0.001) and favorable effects on pain-related quality-of-life scores, with no new safety signals identified. These results reinforce the early and sustained efficacy of Inclisiran and its tolerability in high-risk populations, complementing the findings of the ORION trials. Although this study got published after our cut-off literature search criteria and therefore excluded from the pooled analyses, its results are directionally concordant with our findings and further reinforce the clinical relevance of inclisiran’s durable lipid-lowering efficacy.

Our meta-analysis of 14 RCTs including over 13,000 participants demonstrates consistent and clinically meaningful LDL-C reductions of approximately 45% across ASCVD, HeFH, HoFH, and statin-intolerant populations, while confirming a favorable safety profile with adverse event rates comparable to placebo. Large-scale research including well-powered trials such as ORION 10 and ORION 11 demonstrates that Inclisiran shows strong potential as a potent lipid-lowering agent [44]. The therapeutic effect of Inclisiran remains stable after two doses per year making it stand apart from other lipid treatments and solves two main problems that affect chronic lipid management patients. The lipid trials demonstrate positive effects on apolipoprotein B (apoB) levels together with non-HDL cholesterol and lipoprotein(a) as well as LDL-C reduction [45, 59]. Emerging evidence suggests that reduced visit-to-visit LDL-C variability is independently associated with lower cardiovascular risk. Long-interval therapies such as inclisiran, administered twice yearly, may contribute to more stable LDL-C control, potentially enhancing cardiovascular protection beyond absolute LDL-C reduction [21]. The research evidence indicates that Inclisiran extends its therapeutic benefits beyond basic LDL-C reduction because it demonstrates a wider range of lipid-modulating activities that benefit cardiovascular risk reduction [47, 60]. The safety data collected from these studies reveal a tolerable safety profile with adverse event rates equivalent to those found in placebo-treated patients. Mild transient reactions at the injection site were the most common adverse events but no evidence existed for hepatotoxicity myopathy or immunogenicity. In ORION-1, approximately 5.5% of participants developed anti-drug antibodies (ADAs) against inclisiran [52]. These antibodies were predominantly low-titer, transient, and did not correlate with any attenuation of LDL-C–lowering efficacy. Mechanistically, many ADAs target the GalNAc conjugate moiety or other non-neutralizing epitopes rather than the siRNA itself, and in vitro neutralization assays do not necessarily predict functional loss in vivo [54]. Consistent LDL-C reductions among antibody-positive participants further indicate that these ADAs have minimal clinical impact [61].

From a pharmacological standpoint, RNA interference therapeutics such as inclisiran generally exhibit lower immunogenicity than protein-based therapies, including monoclonal antibodies [62]. Chemical modifications to the siRNA backbone, combined with hepatocyte-targeted delivery via GalNAc conjugation, reduce systemic immune recognition, thereby limiting the likelihood of clinically significant immune responses [63]. As a result, even in the rare presence of ADAs, effective PCSK9 mRNA silencing and robust LDL-C lowering are maintained [32].

Clinically, these observations have important implications for long-term therapy. The low incidence and transient nature of ADAs suggest that routine monitoring for immunogenicity is not required, and the sustained efficacy over extended dosing intervals (biannual administration) supports inclisiran as a practical option for chronic lipid management. Moreover, the negligible impact of ADAs on treatment outcomes reinforces the reliability of inclisiran in both clinical trial and real-world settings, including in patients who may be at higher risk for immunogenic responses. Collectively, these findings underscore the favorable pharmacodynamic and safety profile of inclisiran, supporting its role as a durable and patient-friendly option for achieving LDL-C reduction and potentially mitigating residual cardiovascular risk.

The mechanism of action of Inclisiran represents an innovative approach in lipid management. Through its application of RNA interference (RNAi) technology Inclisiran targets messenger RNA (mRNA) of proprotein convertase subtilisin/kexin type 9 (PCSK9) within hepatocytes to decrease PCSK9 synthesis at the transcriptional level [64, 65]. The therapeutic mechanism of Inclisiran differs from monoclonal antibodies because Inclisiran operates inside cells instead of binding PCSK9 molecules outside cells [14, 66]. Through intracellular PCSK9 reduction Inclisiran enhances liver cell LDL receptor recycling which results in higher receptor density and improved removal of blood LDL particles [9, 67]. Compared with PCSK9 monoclonal antibodies, inclisiran offers distinct advantages and differences. Mechanistically, inclisiran operates intracellularly via RNA interference to suppress PCSK9 mRNA, whereas mAbs neutralize circulating PCSK9 protein. While mAbs achieve slightly higher LDL-C reductions (~ 60%), inclisiran’s twice-yearly dosing may enhance adherence and long-term persistence in real-world settings. Moreover, real-world uptake of PCSK9 mAbs has been limited by frequent injections and cost, whereas inclisiran’s simplified schedule may improve accessibility and implementation in routine lipid management. The intracellular RNAi mechanism allows Inclisiran to deliver prolonged LDL-C reductions because of its three-month and then six-month administration schedule [68, 69]. The studies presented demonstrated sustained maximum LDL-C reduction levels that persisted throughout six months following the last administered dose [25, 42, 70]. The siRNA delivery platform eliminates the chance of immune responses which affect protein-based medications because no anti-drug antibodies (ADAs) developed in trial participants [53, 54]. The pharmacodynamic effects of Inclisiran lead to an extended suppression of plasma PCSK9 levels which directly correlates with significant LDL-C reduction [71]. The dual aspects of PCSK9 suppression and twice-yearly treatment schedule make Inclisiran effective while creating better conditions for patients to adhere to their treatments [47, 72]. The LDL-C lowering capability of Inclisiran reaches approximately 45% which ranks it as one of the strongest lipid-lowering medications but falls short of the 60% reduction seen with PCSK9 monoclonal antibodies evolocumab and alirocumab [47, 73]. The less frequent dosing of Inclisiran might make up for its lower potency since it enhances patient adherence in the long term.

The primary choice of statins works well due to their effectiveness and safety as well as affordability, but they fail to meet LDL-C goals or are poorly tolerated by patients [74–76]. Additional LDL-C reduction from ezetimibe therapy amounts to 15–20% but it often proves insufficient to meet the needs of individuals at high risk [77]. PCSK9 inhibitors have proven their effectiveness but their frequent injections and expensive price have restricted their acceptance by healthcare providers [78, 79]. Inclisiran provides essential therapeutic value to treat patients who cannot take statins as well as those who do not respond to combination therapy and those who need intense lipid management at high cardiovascular risk. The LDL-C reduction results along with its excellent safety profile and simplified administration process indicate Inclisiran may represent a valuable adjunct to existing lipid management strategies, pending confirmation from cardiovascular outcome trials. The research findings demonstrate that Inclisiran functions as a safe and effective treatment for lipid reduction among diverse patient groups [70]. The proven LDL-C lowering effects in both primary and secondary prevention groups make Inclisiran appropriate for patients with ASCVD as well as those at high risk including familial hypercholesterolemia patients and those who cannot take statins. The extended dosing interval which involves two doses per year following an initial loading phase resolves the main challenge in lipid-lowering therapy which is patient adherence. Clinical observations demonstrate that numerous patients stop taking their daily statin medications or PCSK9 antibody injections irregularly which reduces their ability to prevent cardiovascular diseases [44, 80]. Inclisiran’s twice-yearly schedule administered under medical guidance may enhance patient medication adherence which leads to better long-term LDL-C control and decreased cardiovascular complications.

Inclisiran provides safety evidence to make it suitable for elderly patients and those who take many medications or have multiple medical conditions. Treatment choices for lipid-lowering drugs need individual evaluation to make selections that balance patient choices with co-existing medical issues and full cardiovascular profile. The results demonstrate that Inclisiran enables lipid management escalation without the typical safety limitations of present-day lipid therapies so patients who need effective LDL-C reduction can receive treatment in clinical practice. The adoption of therapeutic agents in chronic disease management depends heavily on safety considerations. The safety profile of Inclisiran was equivalent to placebo across the clinical trials which included the RCTs. The most common adverse event was injection-site reactions affecting 5–17% of patients in different studies but these reactions were mild to moderate and short-lived with minimal need to stop treatment [15, 81]. Rare hepatotoxicity or myopathy or renal toxicity events were observed and no concerning trends emerged from routine laboratory tests [82, 83]. The absence of detected anti-drug antibodies in the siRNA platform confirms its low immunogenic nature [52]. The results demonstrate importance because most patients under evaluation had elderly conditions combined with multiple diseases. Inclisiran’s clinical profile enables its safe combination with common lipid-lowering drugs and cardiovascular medicines. The safety data confirm Inclisiran is suitable for prolonged use in standard clinical practice.

The meta-analysis demonstrated substantial interstudy heterogeneity (I² = 90.3%), attributable to both clinical and methodological diversity across the included trials. Variations in patient populations—including those in primary and secondary prevention, heterozygous and homozygous familial hypercholesterolemia (FH), and statin-intolerant cohorts—contributed to the observed variability. Differences in baseline LDL-C levels, comorbid conditions such as diabetes and hypertension, follow-up durations (90–540 days), and concomitant lipid-lowering therapies further influenced effect estimates. The attenuated LDL-C reduction in ORION-5 primarily reflected the inclusion of patients with homozygous FH (HoFH), characterized by markedly reduced or absent LDL receptor (LDLR) activity, which limits responsiveness to PCSK9 inhibition. Funnel plot analysis identified ORION-5 as an outlier, consistent with its small sample size and biologically distinct population. The distinct response in HoFH underscores the mechanistic dependence of inclisiran on residual LDLR function for LDL clearance. Patients with receptor-negative HoFH derive minimal benefit from PCSK9-targeted therapy, whereas receptor-defective HoFH and heterozygous FH (HeFH) patients exhibit clinically meaningful LDL-C reductions [84]. These findings emphasize that the observed heterogeneity arises from biological variability rather than inconsistency in inclisiran efficacy, reinforcing its robust lipid-lowering potential in populations with intact or partially functional LDLR activity. We do not recommend excluding all HoFH patients from PCSK9-targeted therapy. Patients with receptor-defective genotypes may benefit, whereas receptor-negative HoFH often requires LDLR-independent treatments (lomitapide, apheresis) [85]. Genetic characterization should guide therapy selection.

Our study shows positive outcomes, yet multiple important gaps exist in the current field. The research community needs to conduct extensive studies about the long-term effects of inclisiran on cardiovascular outcomes. Real-world studies that examine its effectiveness and adherence rates are necessary to prove that biannual dosing improves patient compliance. The analysis of user patterns alongside the identification of obstacles to implementation together with patient experience outcomes will maximize treatment benefits. Inclisiran requires investigation to establish its effectiveness among pediatric patients with familial hypercholesterolemia together with statin-intolerant patients and those who fail to respond to standard hypercholesterolemia treatments. The combination of Inclisiran with new lipid(a) agents and triglyceride and inflammatory pathway agents could create comprehensive cardiovascular protection benefits. Pharmacogenomic research should develop predictive biomarkers for both therapeutic effects and adverse effects so healthcare providers can tailor treatment strategies.

We used a big pool of high-quality randomized data to provide a complete assessment of Inclisiran safety and effectiveness. The study presents reliable results by using systematic searches and risk of bias evaluations and statistical sensitivity tests. The study faces three major limitations: The patient population across trials showed significant differences which contributed to study heterogeneity and patients received different medications in addition to diverse baseline characteristics and insufficient data about cardiovascular outcomes in the long term. The research contained multiple small studies with weak power to detect effects and wide uncertainty intervals and some selection bias remains despite the funnel plot showing symmetry. The evaluation of cardiovascular advantages requires direct measurement of clinical events, but this study only reports surrogate lipid markers. The exclusion criteria applied might have limited the generalizability of findings by excluding individuals with severe comorbid conditions.

## Conclusions

The present meta-analysis confirms that inclisiran is a potent and durable lipid-lowering agent, achieving substantial reductions in LDL-C across diverse patient populations, including those with established atherosclerotic cardiovascular disease (ASCVD), familial hypercholesterolemia (HeFH and HoFH), statin intolerance, and mixed hyperlipidemia. Through its RNA interference mechanism targeting hepatic PCSK9 synthesis, inclisiran provides sustained suppression of circulating PCSK9, resulting in long-lasting LDL-C reductions with only twice-yearly subcutaneous dosing. This pharmacologic profile addresses two major limitations associated with conventional lipid-lowering therapy: adherence and treatment fatigue, which are critical determinants of long-term LDL-C control and cardiovascular risk mitigation.

The compiled evidence from 14 randomized controlled trials involving more than 13,000 participants demonstrates a consistent LDL-C reduction of approximately 45%, with a favorable safety and tolerability profile. The clinical relevance of these findings lies not only in the magnitude of LDL-C lowering but also in the stability of effect over extended follow-up periods, including open-label extensions such as ORION-8. The twice-yearly dosing regimen represents a significant practical advantage over daily oral therapies and monthly injections, potentially enhancing patient adherence and persistence in real-world settings.

Despite these compelling pharmacological and clinical attributes, it is important to recognize the current evidence gap regarding direct cardiovascular outcomes. No large-scale, fully published randomized trials have yet demonstrated that inclisiran’s LDL-C reduction translates into reductions in major adverse cardiovascular events (MACE). Despite robust LDL-C lowering and safety, cardiovascular outcome benefits of inclisiran remain unproven. The ongoing VICTORION-Outcomes trial will provide definitive evidence on the impact of inclisiran on MACE. Consequently, while the mechanism of action and the established causal relationship between LDL-C lowering and cardiovascular risk provide a strong rationale for potential benefit, formal guideline endorsement as a first-line or core therapy for cardiovascular prevention remains premature. Although VICTORION-Difference was published after our predefined search cut-off, its findings are consistent with our pooled results, demonstrating that biannual Inclisiran achieves rapid and sustained LDL-C lowering while maintaining a favorable safety and tolerability profile. Importantly, the study highlights the benefits of incorporating Inclisiran on top of individualized statin therapy, achieving LDL-C targets in a substantially higher proportion of patients compared with optimized statin therapy alone. These data underscore the potential role of Inclisiran in bridging the residual LDL-C gap in high-risk populations.

Furthermore, while many patients achieve LDL-C targets with optimized statin and ezetimibe therapy, real-world adherence remains suboptimal, particularly over long-term follow-up. Inclisiran’s biannual administration may help overcome this challenge by reducing dosing frequency, potentially improving treatment persistence and lipid control in routine practice. Although inclisiran’s twice-yearly administration may enhance adherence and reduce treatment burden, formal cost-effectiveness analyses are warranted to assess its economic feasibility and potential impact on healthcare resource utilization in routine clinical practice. Nevertheless, long-term observational and health-economic studies are necessary to assess whether these pharmacologic advantages translate into sustained LDL-C lowering, cardiovascular risk reduction, and cost-effective implementation across varied healthcare settings.

In summary, inclisiran represents a scientifically robust and clinically promising adjunct to current lipid-lowering strategies, particularly for patients with residual cardiovascular risk or poor tolerance to standard therapies. Its durable LDL-C reduction, favorable safety profile, and infrequent dosing regimen position it as a transformative therapy for dyslipidemia management. However, integration into clinical guidelines and broader cardiovascular prevention frameworks will require confirmation from forthcoming outcome trials and real-world effectiveness studies, emphasizing the need for evidence-based translation from pharmacologic efficacy to patient-centered cardiovascular benefit.

## Data Availability

All data generated or analyzed during this study are included within the manuscript.

## Abbreviations

siRNA: Small Interfering Ribonucleic Acid
LDL-C: Low-Density Lipoprotein Cholesterol
PCSK9: Proprotein Convertase Subtilisin/Kexin Type 9
RCTs: Randomized Controlled Trials
CV: Cardiovascular
ASCVD: Atherosclerotic Cardiovascular Disease
FH: Familial Hypercholesterolemia
LDLR: Low-Density Lipoprotein Receptor
RNAi: RNA Interference
mRNA: Messenger Ribonucleic Acid
ORF: Open Reading Frame
ApoB: Apolipoprotein B
HMGCR: 3-Hydroxy-3-Methylglutaryl-Coenzyme A Reductase
Lp(a): Lipoprotein(a)
CRP: C-Reactive Protein
T2DM: Type 2 Diabetes Mellitus
CVD: Cardiovascular Disease
FDA: Food and Drug Administration
EMA: European Medicines Agency
NICE: National Institute for Health and Care Excellence
PRISMA: Preferred Reporting Items for Systematic Reviews and Meta-Analyses
PICOS: Population, Intervention, Comparator, Outcome, Study Design
CENTRAL: Cochrane Central Register of Controlled Trials
CI: Confidence Interval
SD: Standard Deviation
MD: Mean Difference
I²: I-squared statistics (for heterogeneity)
CoV: Coefficient of Variation
LLOQ: Lower Limit of Quantification
GLP: Good Laboratory Practice
MedDRA: Medical Dictionary for Regulatory Activities
AE: Adverse Event
SAE: Serious Adverse Event
HCRU: Healthcare Resource Utilization
LDLR mRNA: Low-Density Lipoprotein Receptor Messenger RNA
LDL: Low-Density Lipoprotein
HDL: High-Density Lipoprotein
TG: Triglycerides
TC: Total Cholesterol
AHA: American Heart Association
ESC: European Society of Cardiology
SPC: Summary of Product Characteristics
SDG: Sustainable Development Goals
AC: Active Comparator
DBP: Diastolic Blood Pressure
SBP: Systolic Blood Pressure
CVOT: Cardiovascular Outcomes Trial
GRADE: Grading of Recommendations, Assessment, Development and Evaluations
RISC: RNA-Induced Silencing Complex
GalNAc: N-acetylgalactosamine
ASGPR: Asialoglycoprotein Receptor
